# Chronic treatment with corticosterone increases the number of tyrosine hydroxylase-expressing cells within specific nuclei of the brainstem reticular formation

**DOI:** 10.3389/fnana.2022.976714

**Published:** 2022-10-28

**Authors:** Carla Letizia Busceti, Domenico Bucci, Mariarosaria Scioli, Paola Di Pietro, Ferdinando Nicoletti, Stefano Puglisi-Allegra, Michela Ferrucci, Francesco Fornai

**Affiliations:** ^1^Department of Molecular Pathology, IRCCS Neuromed, Pozzilli, Italy; ^2^Department of Physiology and Pharmacology, Sapienza University of Rome, Rome, Italy; ^3^Department of Translational Research and New Technologies in Medicine and Surgery, University of Pisa, Pisa, Italy

**Keywords:** area postrema, retrorubral field, brainstem, Cushing's syndrome, catecholamines, glucocorticoids, glucose tolerance

## Abstract

Cushing's syndrome is due to increased glucocorticoid levels in the body, and it is characterized by several clinical alterations which concern both vegetative and behavioral functions. The anatomical correlates of these effects remain largely unknown. Apart from peripheral effects induced by corticosteroids as counter-insular hormones, only a few reports are available concerning the neurobiology of glucocorticoid-induced vegetative and behavioral alterations. In the present study, C57 Black mice were administered daily a chronic treatment with corticosterone in drinking water. This treatment produces a significant and selective increase of TH-positive neurons within two nuclei placed in the lateral column of the brainstem reticular formation. These alterations significantly correlate with selective domains of Cushing's syndrome. Specifically, the increase of TH neurons within *area postrema* significantly correlates with the development of glucose intolerance, which is in line with the selective control by *area postrema* of vagal neurons innervating the pancreas. The other nucleus corresponds to the retrorubral field, which is involved in the behavioral activity. In detail, the retrorubral field is likely to modulate anxiety and mood disorders, which frequently occur following chronic exposure to glucocorticoids. To our knowledge, this is the first study that provides the neuroanatomical basis underlying specific symptoms occurring in Cushing's syndrome.

## Introduction

Cushing's syndrome is due to increased glucocorticoid levels in the body, and it is characterized by several clinical alterations which concern vegetative and behavioral functions (Starkman et al., 1992). For instance, altered metabolism (including diabetes), increased blood pressure, sleep disorders, increased feeding, aggressiveness, anxiety, and psychosis have been described in patients suffering from Cushing's syndrome (Krieger and Glick, 1974; Shipley et al., 1992; Ntali et al., 2015). Also, increased feeding is often reported in patients with increased glucocorticoid levels, which results in an increase in body weight (Chanson and Salenave, 2010).

The anatomical correlates of these effects remain largely unknown. Apart from peripheral effects induced by corticosteroids as counter-insular hormones, only a few reports allow us to hypothesize which neurobiology underlies glucocorticoids-induced vegetative and behavioral alterations. Among these, a seminal article published by Sloviter et al. (1989) showed that adrenalectomy in rats causes profound hippocampal electrophysiological alterations and a nearly complete loss of granule cells in the hippocampal dentate gyrus. These authors demonstrated that corticosterone replacement rescues electrophysiological responses and prevents cell loss of hippocampal dentate granule cells in adrenalectomized rodents. This suggests that glucocorticoids play a fundamental role in maintaining the structural integrity of the normal adult hippocampus (Sloviter et al., 1989). However, it is unlikely that multiple metabolic and behavioral alterations induced by an excess of glucocorticoids may be entirely generated by hippocampal dysfunctions. Thus, it remains to be elucidated which other brain regions may contribute to increased feeding, altered glucose tolerance, anxiety, increased blood pressure, and alterations in the sleep pattern, which characterize Cushing's syndrome. Due to the seminal role of the brainstem reticular formation in promoting the sleep-waking cycle (Moruzzi and Magoun, 1949), alertness, and anxiety, as well as the specific vegetative control of the cardiovascular system and specific abdominal organs, this area deserves specific investigations. The reticular formation contains nuclei that are responsible for sleep-waking cycle, anxiety, aggressiveness, as well as blood pressure control and, in the case of area postrema (AP), specific control of pancreatic secretion (Loewy et al., 1994). The recruitment of these domains in Cushing's syndrome questions whether glucocorticoid may alter the brainstem reticular formation. Among reticular nuclei, these effects are mainly controlled by the lateral, TH-positive column of reticular nuclei (Bucci et al., 2017, 2018). In line with this, in a recent article, we indicated a selective increase of catecholamine cells placed in the caudal part of the lateral column of the brainstem reticular formation. This evidence was limited to investigations carried out in organotypic cell cultures following corticosterone incubation (Busceti et al., 2019). Here, aiming at translating these effects into system neurobiology, we investigated *ex vivo*, in the whole brain, which nuclei of the brainstem reticular formation may be altered concomitantly with the occurrence of glucose intolerance and increased body weight following chronic exposure to corticosteroids in mice. Such a project encompassing a plethora of behavioral and vegetative domains cannot be solved in a single research article. Due to the recent discovery of a prominent role of AP in controlling the subdivision of the vegetative nervous system innervating the pancreas, the present study mostly focused on correlating altered TH expression in AP with altered glucose tolerance, which typically features Cushing's syndrome.

## Materials and methods

### Animals

For these experiments, we used 8-weeks-old C57Bl/6J male mice (*N* = 18) (Charles River, Calco, LC, Italy). All animals were maintained under controlled conditions (room temperature = 22°C; humidity = 40%) on a 12-h light-dark cycle with food and water *ad libitum*.

### Experimental strategy

**Figure 1:**
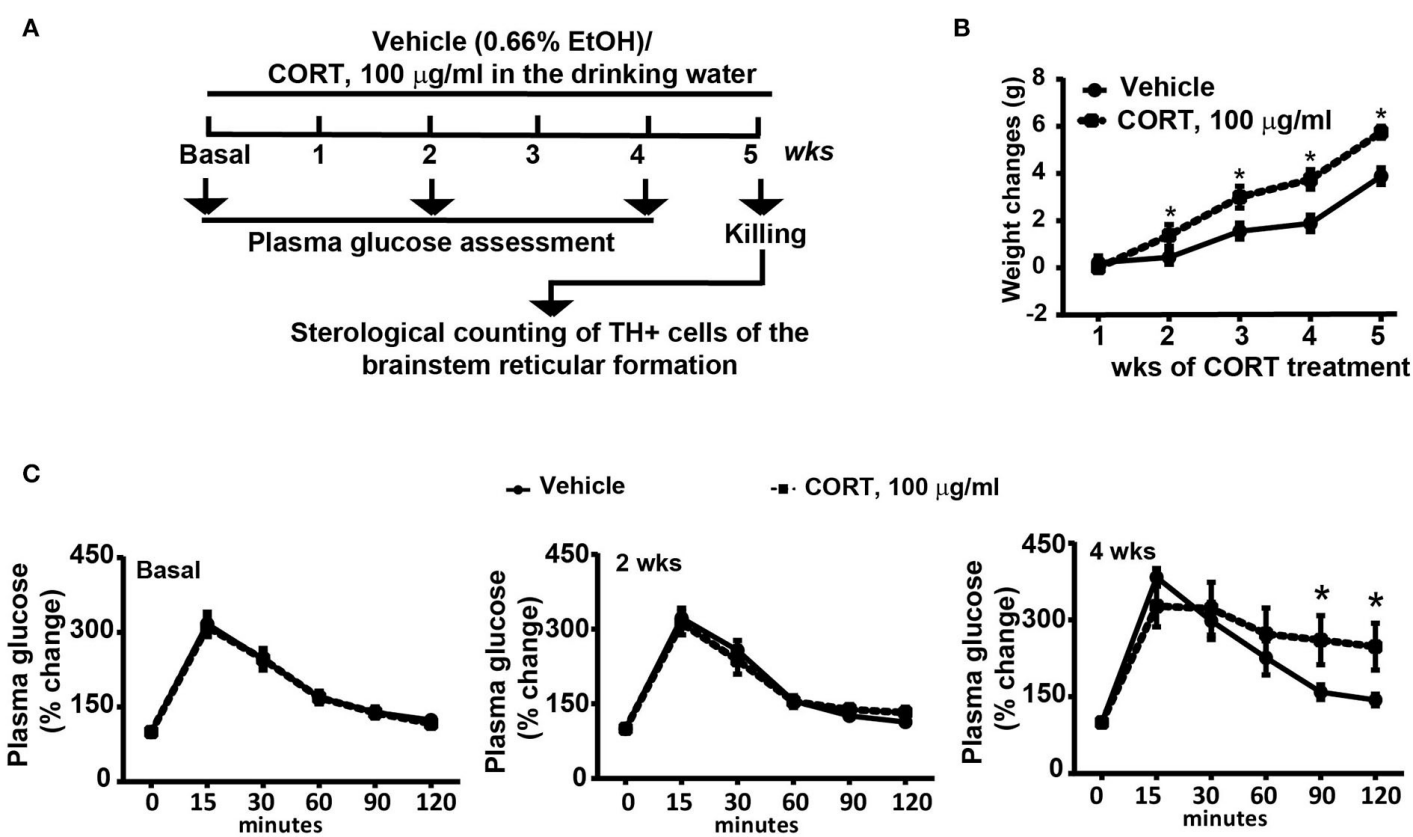
Chronic treatment with corticosterone induced in mice higher body weight and glucose intolerance. **(A)** Experimental strategy used to induce an animal model of exogenous glucocorticoid excess in mice (*N* = 9 per group). **(B)** Body weight changes in response to treatment for 5 weeks with vehicle [0.66% of ethanol (Et-OH)] or corticosterone (CORT, 100 μg/ml in the drinking water). Values are means +/– S.E.M. of 9 mice per group. **(C)** Glucose tolerance curve in mice before (Basal) or following 2 weeks or 4 weeks of chronic treatment with vehicle or CORT (values are means +/– S.E.M. of 8 mice per group with the exclusion of 1 mouse per group as not-responder to the glucose bolus). *p < 0.05, Two-way RM Anova.

C57Bl/6J male mice (*N* = 9) were chronically administered with corticosterone for 5 weeks (Sigma Aldrich, MI, Italy, code: C-2505) in the drinking water (normal drinking water was replaced with a 0.66% ethanol solution containing 100 μg/mL corticosterone). Based on the daily water intake, the daily dose of corticosterone ranges between 1.5 mg/Kg and 2 mg/Kg. Vehicle-treated mice (*N* = 9) were treated for 5 weeks with a 0.66% ethanol solution in the drinking water. Solutions were freshly prepared. Body weight changes were monitored weekly during the treatment period (5 weeks, Figure 1A). All mice were assessed for glucose tolerance under basal conditions and after 2 or 4 weeks of chronic treatment with corticosterone (Figure 1A), body weight was monitored. At the end of the treatment, all mice were killed and dissected brains were used for the immunohistochemical analysis of TH-positive cells in the whole rostro-caudal extension of the brainstem reticular formation (Figure 1A). All anatomical points of reference were indicated according to the atlas of Paxinos and Franklin (2001) for mice.

### Glucose tolerance test

A glucose solution (20% in 0.9% NaCl) was administered by intraperitoneal (i.p.) injection (100 μl/10 g body weight). Blood glucose was measured at five time points (15, 30, 60, 90, and 120 min after i.p. injection of glucose) during the following 4 h. Blood samples were obtained by a small incision on the paw, and glucose levels were measured by using the blood glucometer One Touch Vita (Johnson & Johnson, NY, USA).

### Immunohistochemistry

Dissected brains were fixed overnight at 4°C in Carnoy's solution (60% ethanol, 10% acetic acid, and 30% chloroform with a ratio of fixing solution to the tissue of 20:1 in weight). After fixing the tissue, brain samples were embedded in paraffin and cut with a rotative microtome (Leica, Wetzlar, Germany, code: RM 2245) to obtain 20 μm thick sections. These slices were sampled along the whole rostro-caudal extent of the brainstem reticular formation. Tissue sections were incubated overnight with a monoclonal mouse anti-TH primary antibody (1:100; Sigma Aldrich, code: T1299) and then for 10 min with a secondary biotin-coupled anti-mouse secondary antibody (1:400; Vector Laboratories, Burlingame, CA, USA code: BA-2000). 3,3-Diaminobenzidine tetrachloride (Sigma Aldrich, code: D4293-50set) was used for detection. Negative control was performed without incubation with primary antibody.

### Sampling method

Stereological analysis for all catecholaminergic nuclei was carried out on serial coronal slices sampled every 160, 80, and 40 μm for substantia nigra pars compacta (SNC), ventral tegmental area (VTA), and all other nuclei counted, respectively. This sampling paradigm was established based on a pilot analysis carried out by using different inter-slice intervals (160, 80, and 40 μm). This allows us to obtain a CE value which is ~ 0.1.

As shown in Table 1, when sampling with high inter-slice intervals, there is relatively high coefficient of error (CE) values for most nuclei assessed, which can be reduced by reducing the inter-slice interval. This condition is intrinsically dependent on the low density of scattered TH-positive cells within most catecholaminergic nuclei of the brainstem reticular formation. Consistently, the CE values cannot be less than 0.1 as in the case of more densely packed neuron nuclei (West et al., 1996; Lewitus et al., 2012; Dell et al., 2016).

**Table 1:** Pilot analysis for stereological cell counting of catecholaminergic nuclei of the mouse brainstem reticular formation.

**Area**	**Rostro–caudal extension**	**Sampling interval**	**Counted levels**	**Total number**	**CE**
SNC	1,260 μm (Br −2.54/−3.80)	Every 160 μm	8	14,443	0.083
		Every 80 μm	15	14,226	0.059
		Every 40 μm	29	14,864	0.041
VTA	960 μm (Br −2.92/−3.88)	Every 160 μm	6	9,670	0.115
		Every 80 μm	11	9,955	0.080
		Every 40 μm	21	9,829	0.057
A8	320 μm (Br −3.80/−4.12)	Every 160 μm	3	2,605	0.229
		Every 80 μm	5	2,549	0.164
		Every 40 μm	9	2,575	0.116
PAG	480 μm (Br −3.80/−4.28)	Every 160 μm	4	3,050	0.200
		Every 80 μm	7	2,842	0.147
		Every 40 μm	13	2,999	0.101
PB	480 μm (Br −4.84/−5.32)	Every 160 μm	4	1,724	0.235
		Every 80 μm	7	1,771	0.164
		Every 40 μm	13	1,798	0.113
A7	320 μm (Br −5.00/−5.32)	Every 160 μm	3	1,329	0.333
		Every 80 μm	5	1,376	0.242
		Every 40 μm	9	1,296	0.169
A6	480 μm (Br −5.34/−5.82)	Every 160 μm	4	3,983	0.171
		Every 80 μm	7	3,853	0.119
		Every 40 μm	13	3,862	0.084
A5	640 μm (Br −5.34/−5.98)	Every 160 μm	5	469	0.577
		Every 80 μm	9	499	0.333
		Every 40 μm	17	480	0.258
NTS	340 μm (Br −7.06/−7.40)	Every 160 μm	3	1,389	0.288
		Every 80 μm	5	1,374	0.208
		Every 40 μm	9	1,423	0.141
C2/A2	1440 μm (Br −6.36/−7.8)	Every 160 μm	9	671	0.208
		Every 80 μm	17	635	0.147
		Every 40 μm	33	648	0.095
C1/A1	1440 μm (Br −6.36/−7.8)	Every 160 μm	9	1,013	0.171
		Every 80 μm	17	981	0.115
		Every 40 μm	33	1,026	0.079
AP	440 μm (Br −7.32/−7.76)	Every 160 μm	3	1,432	0.242
		Every 80 μm	5	1,491	0.141
		Every 40 μm	9	1,456	0.113

The rostro-caudal extension, the different inter-slice intervals used for quantification (160 μm, 80 μm, and 40 μm), the respective number of counted levels, the total number of TH-positive cells counted and the coefficient of error (CE) values are shown for all catecholaminergic nuclei assessed (SNC, VTA, A8, PAG, A7, PB, A6, A5, NTS, C2/A2, C1/A1, and AP).

### Stereology

Stereological counting of TH-positive cells was carried out by using a microscope Zeiss Axio Imager M1 (Zeiss, Wetzlar, Germany) associated with the software Image Pro-Plus 6.2 for Windows (vers. 6.2.1.491, Media Cybernetics, inc., Rockville, MD) equipped with a specific Macro (obtained by Immagine and Computer, Italy, MI) created *ad hoc* to perform the Optical Dissector technique. This Macro allows the operator to obtain an unbiased and optimized stereological cell count, according to King et al. (2002). All the areas of interest were identified and outlined at 2.5 × magnification. TH-positive cells were then counted at 100X magnification (numerical aperture 1.3) as previously described (King et al., 2002) by using a different dissector grid depending on the volume of the area to be analyzed. At the end of the procedure, a data sheet was produced containing all the data necessary to obtain the number of cells.

The total estimation of cell numbers (*N*) was calculated by using the following equation:


N = Σ(Q-) x 1/ssf x 1/asf x 1/tsf

Where *ssf* is the “section sampling fraction,” *asf* is the “area sampling fraction,” *tsf* if the “thickness sampling fraction” (thickness of the tissue divided by the dissector height), and Σ*Q*- is the total number of cells counted within the dissector.

The “Section Sampling Fraction” (*ssf*) is represented by the number of regularly spaced sections used for counts divided by the total number of sections used to collect the entire structure of interest. To sample the whole brainstem area, we collected 261 sections of 20 μm (covering the full extent of the area which is 5,220 μm). Sections were sampled at a ratio of 1:8, 1:4, and 1:2 (*ssf*) for SNC, VTA, and all other nuclei assessed, respectively.

The “Area Sampling Fraction” (*asf*) represents the area between dissectors, that is, the ratio between the area of counting frames and the Area of Interest (AOI).

The remaining value is *tsf* , the “Thickness Sampling Frequency,” that is, the height sampling fraction and it is calculated as the ratio between the height of the counting frame and the thickness of the tissue. This value is calculated by our system in each counting frame. The Coefficient of error (CE) was calculated according to King et al. (2002).

### Statistical analysis

Data are given as the mean ± SEM with statistical significance defined by *p* < 0.05. Statistical analyses were performed as follows: *(i)* Unpaired two-tailed Student's *t*-test (**Figures 8A–C, 9A–H, 10A–D**); *(ii)* Two-way RM ANOVA followed by Fisher's LSD (Figures 1B,C); and *(iii)* Pearson correlation test (**Figures 11A–L, 12A–L**). GraphPad Prism (Ver 5.01 GraphPad Software, Inc. La Jolla, CA, USA) statistical software was used for analysis.

## Results

### Chronic administration of corticosterone increases the number of catecholamine cells in the whole mouse brainstem reticular formation

**Figure 2:**
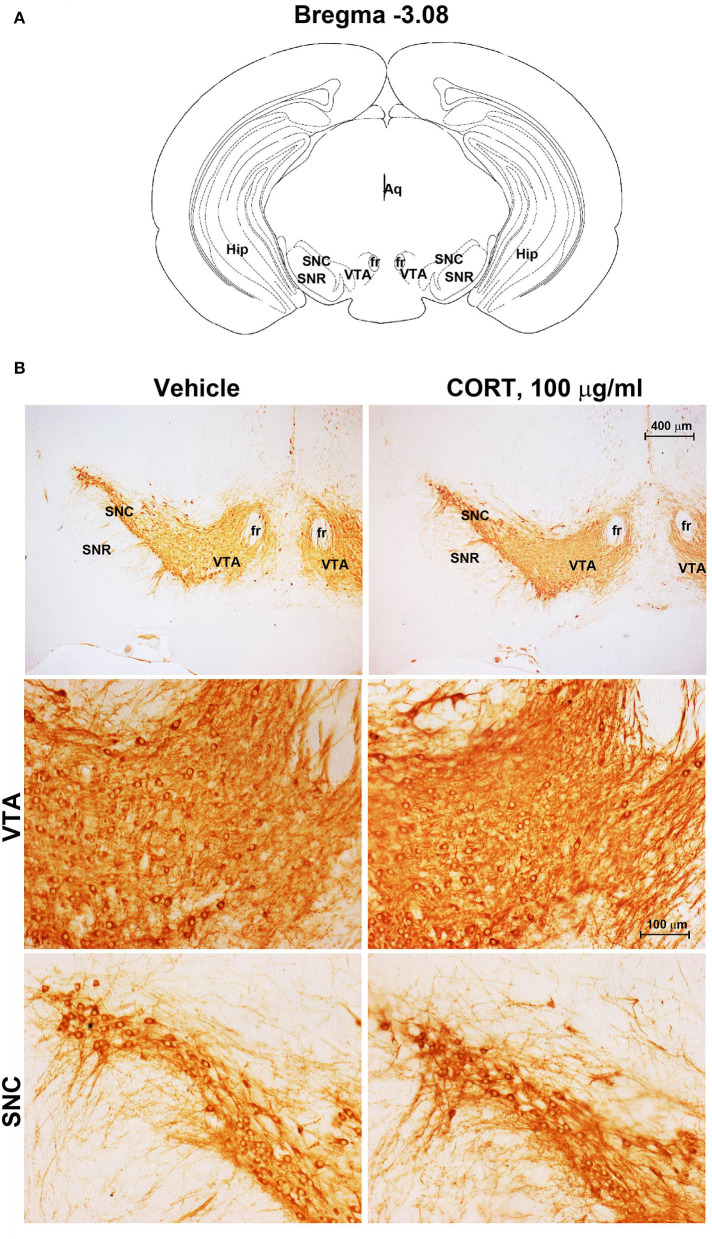
Chronic treatment with corticosterone does not change TH-immunoreactivity in the substantia nigra pars compacta and ventral tegmental area. **(A)** Schematic picture modified from the atlas of Paxinos and Franklin (2001) for mice showing the anatomical location of the catecholaminergic nuclei in substantia nigra pars compacta (SNC) and ventral tegmental area (VTA) at bregma level −3.08. Aq, Aqueduct of Sylvius; fr, fasciculus retroflexus; Hip, hippocampus; SNR, substantia nigra pars reticulata. **(B)** Representative images of TH-immunoreactive cells in SNC and VTA of mice subjected to chronic treatment with vehicle (ethanol 0.66%) or corticosterone (CORT, 100 μg/ml).

**Figure 3:**
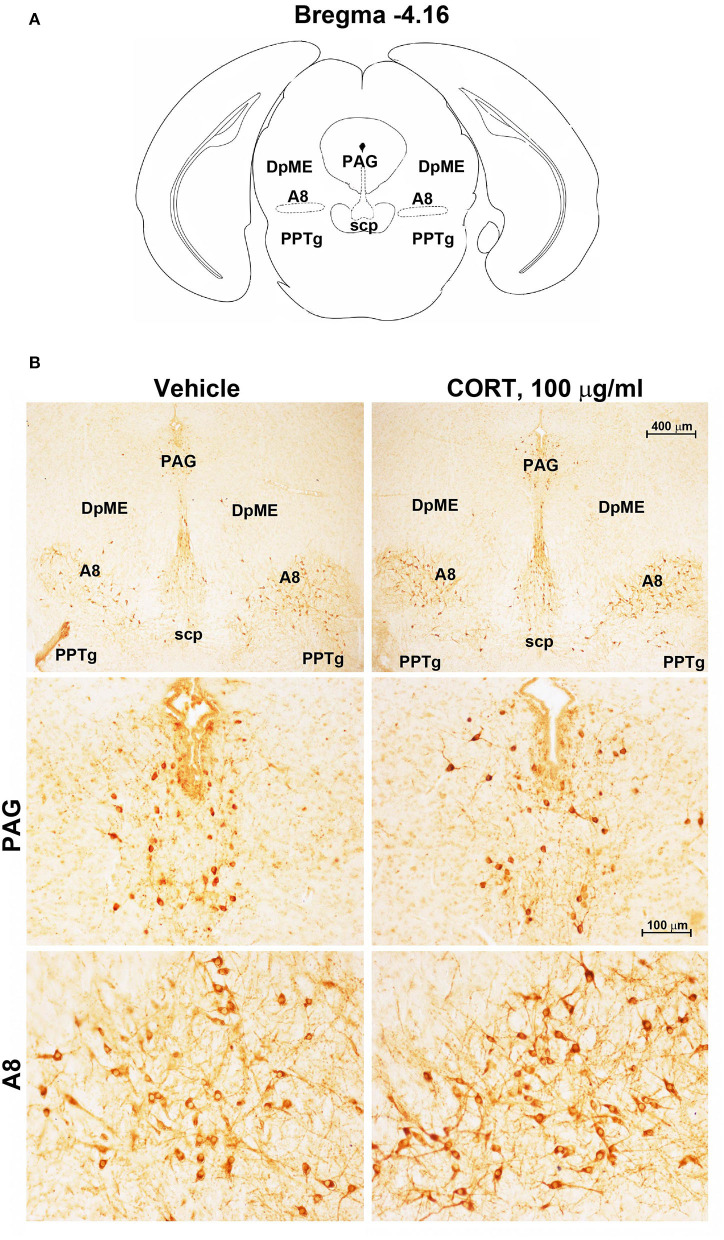
Chronic treatment with corticosterone increases TH-immunoreactivity in the retrorubral field (RRF). **(A)** Schematic picture modified from the atlas of Paxinos and Franklin (2001) for mice showing the anatomical location of the catecholaminergic nuclei in the peri-aqueductal gray (PAG) and retrorubral field (A8) at bregma level −4.16. DpME, deep mesencephalic nucleus; scp, superior cerebellar peduncle; PPTg, pedunculopontine tegmental nucleus. **(B)** Representative images of TH-immunoreactive cells in PAG and A8 of mice subjected to chronic treatment with vehicle (ethanol 0.66%) or corticosterone (CORT, 100 μg/ml).

**Figure 4:**
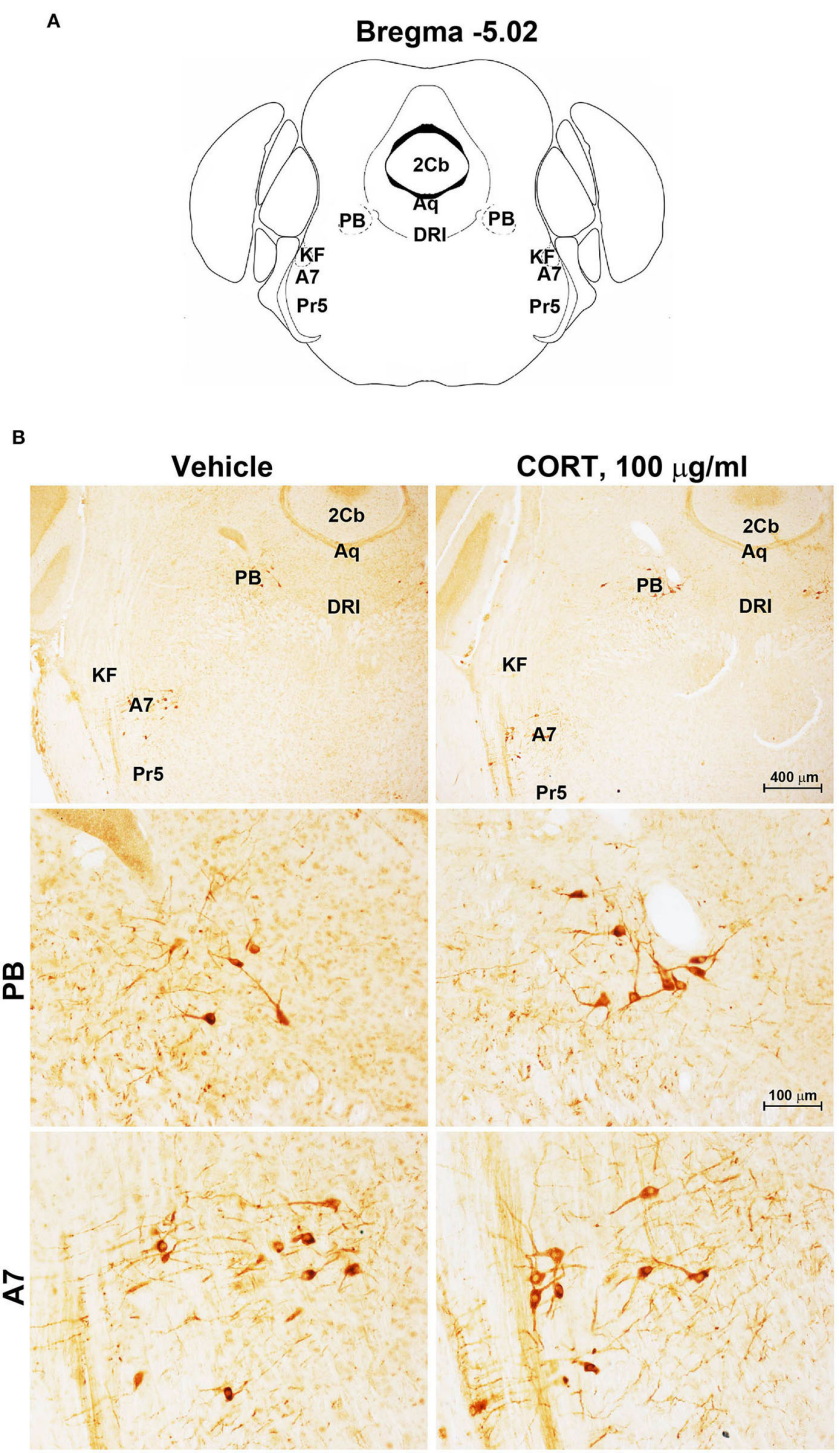
Chronic treatment with corticosterone does not change TH-immunoreactivity in the parabrachial nucleus and lateral lemniscus. **(A)** Schematic picture modified from the atlas of Paxinos and Franklin (2001) for mice showing the anatomical location of the catecholaminergic nuclei in the parabrachial nucleus (PB) and lateral lemniscus (A7) at bregma level −5.02. Aq, Aqueduct of Sylvius; 2Cb, Second Cerebellar lobule; DRI, dorsal raphe nucleus, interfascicular part; KF, Kölliker-Fuse nucleus; Pr5, principal sensory trigeminal nucleus. **(B)** Representative images of TH-immunoreactive cells in PB and A7 of mice subjected to chronic treatment with vehicle (ethanol 0.66%) or corticosterone (CORT, 100 μg/ml).

**Figure 5:**
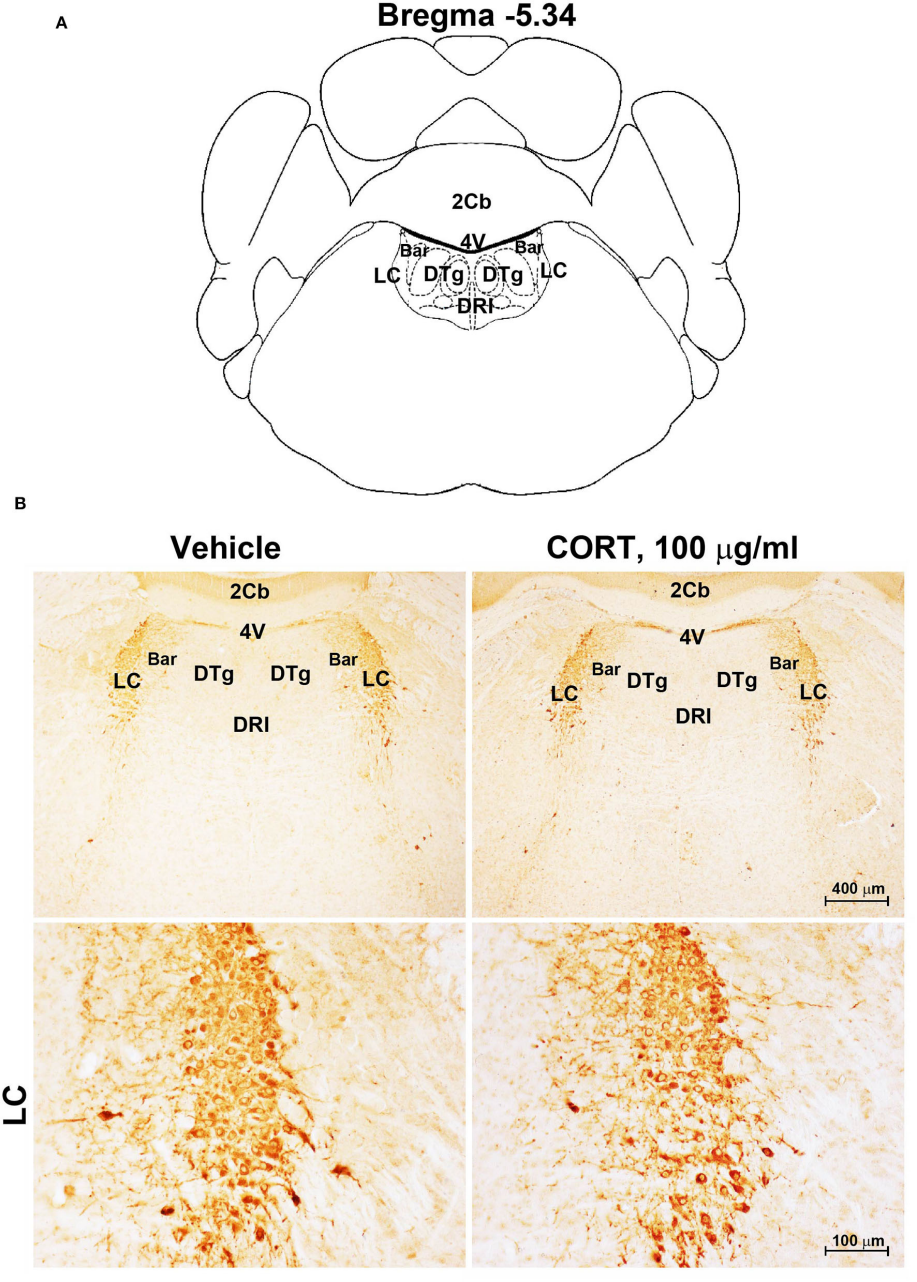
Chronic treatment with corticosterone does not induce variations in TH-immunoreactivity in the *locus coeruleus*. **(A)** Schematic picture modified from the atlas of Paxinos and Franklin (2001) for mice showing the anatomical location of *Locus Coeruleus* (LC) at the bregma level −5.34. Bar, Barrington's nucleus; 2Cb, Second Cerebellar lobule; DRI, dorsal raphe nucleus, interfascicular part; DTg, dorsal tegmental nucleus; 4V, 4^th^ ventricle; **(B)** Representative images of TH-immunoreactive cells in the LC of mice subjected to chronic treatment with vehicle (ethanol 0.66%) or corticosterone (CORT, 100 μg/ml).

**Figure 6:**
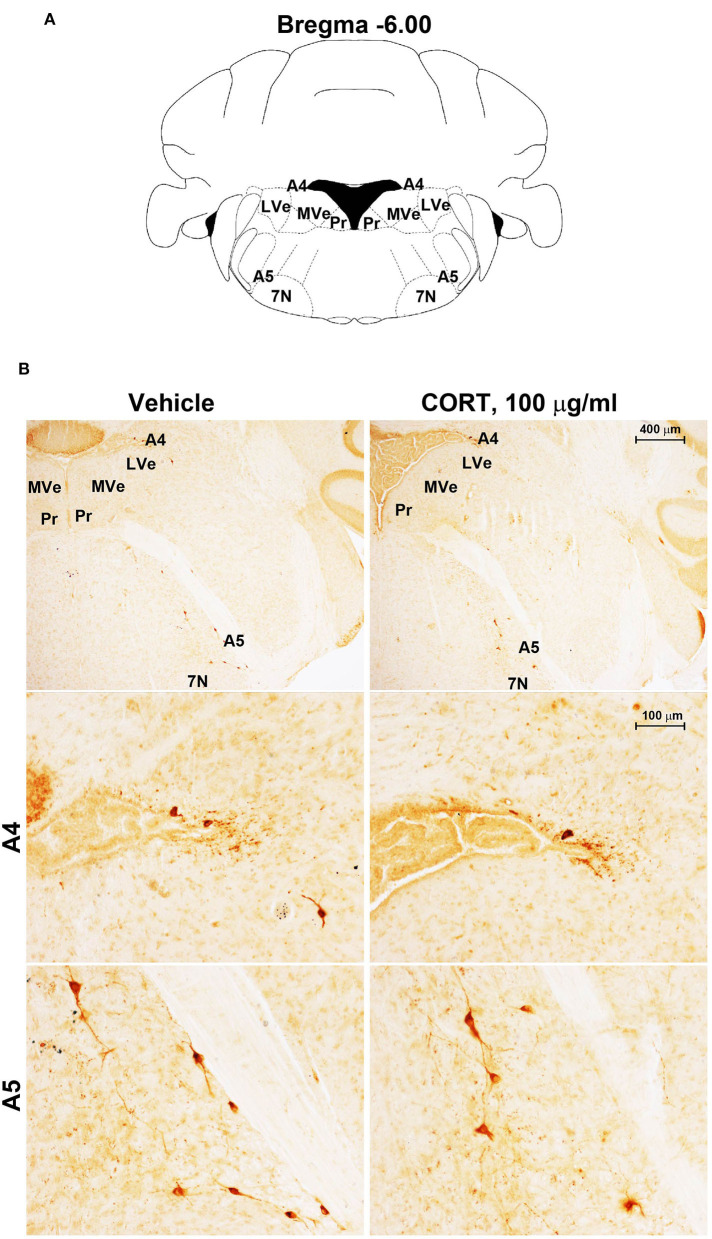
Chronic treatment with corticosterone does not change TH-immunoreactivity in the A5 catecholamine nucleus. **(A)** Schematic picture modified from the atlas of Paxinos and Franklin (2001) for mice showing the anatomical location of the A5 catecholamine nucleus at the bregma level −6.00. 7N, facial nucleus; Pr, prepositus nucleus; LVe, lateral vestibular nucleus; MVe, medial vestibular nucleus. **(B)** Representative images of TH-immunoreactive cells in A4 and A5 catecholamine nuclei in the brainstem reticular formation of mice subjected to chronic treatment with vehicle (ethanol 0.66%) or corticosterone (CORT, 100 μg/ml).

**Figure 7:**
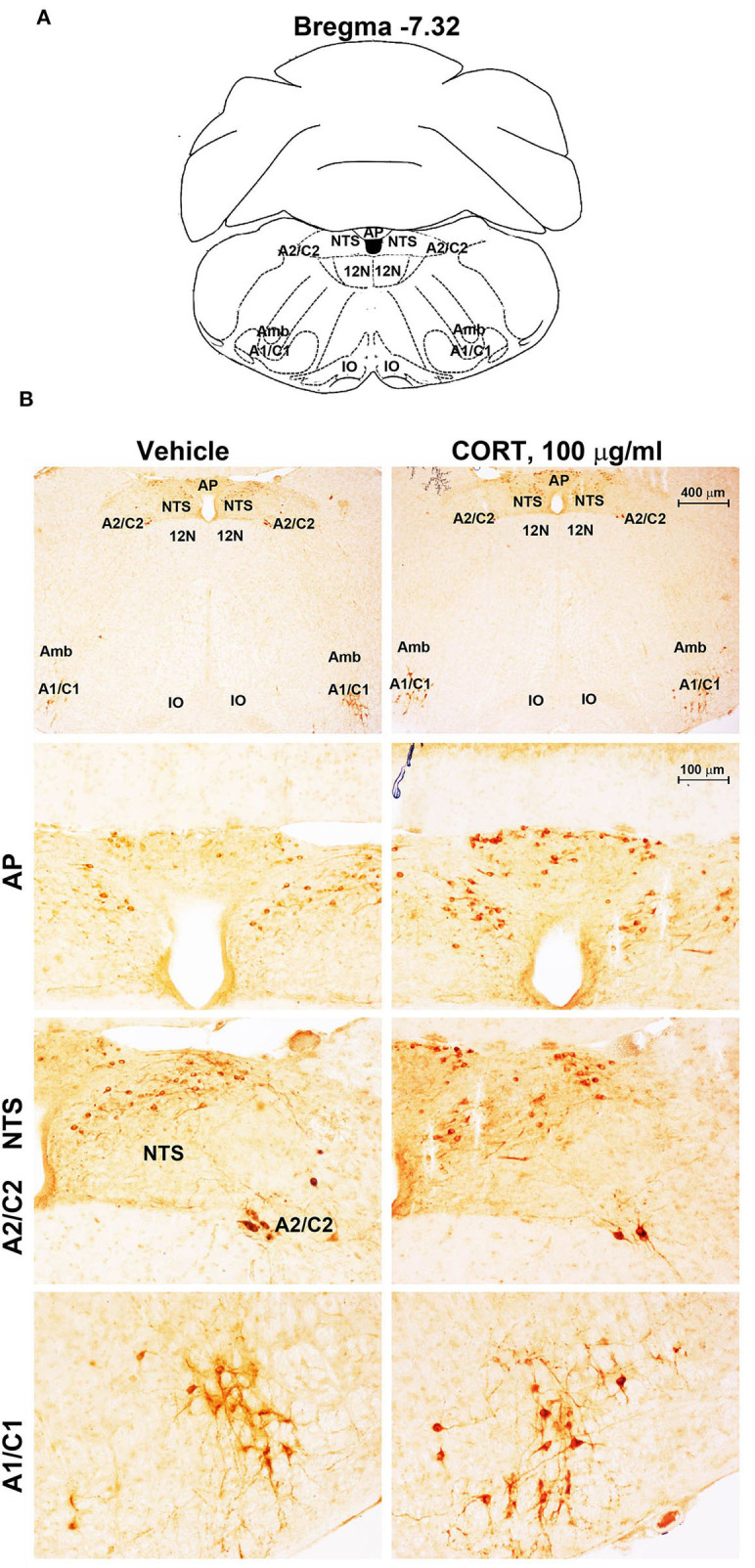
Chronic treatment with corticosterone increases TH-immunoreactivity in the area postrema (AP) of brainstem reticular formation. **(A)** Schematic picture modified from the atlas of Paxinos and Franklin (2001) for mice showing the anatomical location of the rostral ventrolateral medulla (C1/A1), the dorsomedial nucleus of ala cinerea (C2/A2), the nucleus of the solitary tract (NTS), and the area postrema (AP) at the bregma level −7.32. Amb, nucleus Ambiguus; IO, inferior olive; 12N, hypoglossal nucleus. **(B)** Representative images of TH-immunoreactive cells AP, NTS, A2C2, and A1/C1 catecholamine nuclei in the brainstem reticular formation of mice subjected to chronic treatment with vehicle (ethanol 0.66%) or corticosterone (CORT, 100 μg/ml).

Chronic administration of corticosterone (100 μg/mL in the drinking water for 5 weeks, Figure 1A) to C57Bl/6J male mice induces a progressive increase in body weight (Figure 1B) and occurrence of glucose intolerance 4 weeks after treatment (Figure 1C). Serial sections of the rostral and caudal brainstem were considered to include 12 catecholamine nuclei of the brainstem reticular formation. (i) The dopamine-containing mesencephalic nuclei: A8 (also known as retrorubral field, RRF) (Bregma −3.8/Bregma −4.12); A9 (SNC) (Bregma −2.54/Bregma −3.80); and A10 (VTA) (Bregma −2.92/Bregma −3.88). (ii) TH-positive cells inside the peri-aqueductal gray (PAG) (Bregma −3.8/Bregma −4.28); (iii) the pontine parabrachial nucleus (PB) (Bregma−4.84/Bregma −5.32); (iv) the A7 nucleus (nucleus of lateral lemniscus) (Bregma −5.00/Bregma −5.32); (v) the big pontine noradrenergic nucleus A6 (locus coeruleus, LC) (Bregma −5.34/Bregma −5.82); (vi) the A5 nucleus (Bregma −5.34/Bregma −5.98); (vii) the rostral ventrolateral medulla C1/A1 (Bregma −6.36/Bregma −7.8); (viii) the dorsomedial nucleus of ala cinerea, C2/A2 (Bregma −6.36/Bregma −7.8); (ix) TH-positive cells inside the nucleus of the solitary tract (NTS) (Bregma −7.06/Bregma −7.4); and *(x)* the area postrema (AP) (Bregma −7.32/Bregma −7.76) (representative pictures of Figures 2–7). This provides a scenario encompassing the lateral column of the brainstem reticular formation ranging from the SNC down to the AP (Figures 2, 7, respectively). These representative pictures aim to provide the anatomical background of brainstem reticular catecholamine nuclei. It is remarkable that, as shown in representative Figure 6, even the undefined A4 region could be well visualized in these slices. When considering altogether these 12 nuclei, no consistent difference was noticeable by comparing corticosterone- and vehicle-treated mice. Only in the most extreme regions (RRF and AP), placed at the rostral and caudal pole of the brainstem reticular formation, respectively, a difference in TH immunostaining was evident (compare representative pictures of Figures 3, 7 as well as **Figures 9C, 10D**). This was substantiated by the total cell count (**Figures 9C, 10D**).

**Figure 8:**
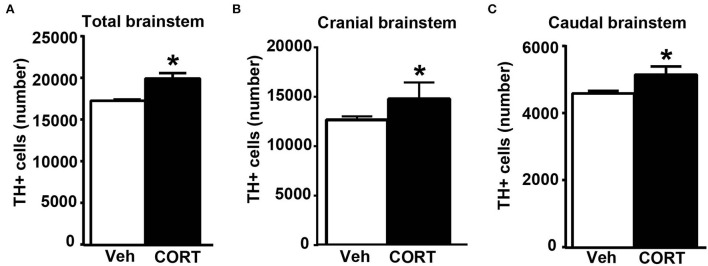
Chronic treatment with corticosterone increases the number of catecholamine cells in the pons and medulla oblongata. **(A)** Stereological counting of TH-immunoreactive cells into the whole rostro-caudal extension of the pons and medulla oblongata (from Bregma = −3.8 to Bregma = −7.64 without including the rostral midbrain) of mice subjected to chronic treatment with corticosterone (CORT, 100 μg/ml) and their control vehicle (ethanol 0.66%) mice. The number of TH-positive cells in catecholamine nuclei of the cranial brainstem (including A8, PAG, A7, PB, A6, and A5 catecholamine nuclei) or caudal brainstem (including NTS, C2/A2, C1/A1, and AP) of mice subjected to chronic treatment with vehicle or corticosterone is shown in **(B,C)**, respectively. Values are means +/– S.E.M. of nine (vehicle) and eight (CORT) mice per group (one mouse in the group of corticosterone-treated mice was eliminated as a non-responder). *p < 0.05 Unpaired two-tailed Student *t*-test.

Consistently with our previous findings obtained in organotypic mouse brainstem cultures (Busceti et al., 2019), stereological counting provides evidence for an increased number of TH-immunopositive cells in the whole rostro-caudal extension of the pons and medulla oblongata (from Bregma = −3.8 to Bregma = −7.64 without including the rostral midbrain) of mice treated with corticosterone compared with control vehicle-treated mice (Figure 8A).

To assess the specific placement of increased TH-positive cells, which occurred following corticosterone administration *in vivo*, a stereological quantification was carried out by differentiating the cranial (Bregma −3.8/Bregma −5.82) from the caudal (Bregma −6.36/Bregma −7.64) part of the brainstem. Differing from *in vitro* data reporting an increase of TH in the caudal nuclei only (Busceti et al., 2019), the present investigation carried out *ex vivo* indicates that corticosterone-induced increase in the number of TH-immunopositive cells similarly occurs within cranial and caudal parts of the mouse brainstem (Figures 8B,C, respectively).

This suggests that, when administered *in vivo*, corticosterone significantly increases TH immunostaining in multiple brainstem regions compared with its effects in isolated brainstem slices. In detail, when counted *ex vivo*, the increase in the rostral brainstem overalls the increase in the caudal brainstem and both express representatively the increase in TH-positive cell bodies, which was measured in the whole brainstem (Figure 8). The previous study using organotypic cell cultures left the increase of TH immunopositive neurons in the caudal brainstem non-defined since stereology could not be carried out and the increase was roughly attributed to catecholamine cell groups within the lower medulla. In contrast, the present study, which is carried out *ex vivo* identifies specifically the caudal appendix of this region, the AP, as the specific part where the increase in TH takes place. For what concerns the rostral brainstem, the specific nucleus of RRF owns the increase in TH immunostaining.

### In the cranial brainstem, TH-immunopositive cells increase significantly within the retrorubral field

To obtain a more detailed anatomical regional mapping of corticosterone-induced increase in the number of TH-positive cells, a detailed stereological analysis was carried out for each catecholamine nucleus of the brainstem reticular formation.

**Figure 9:**
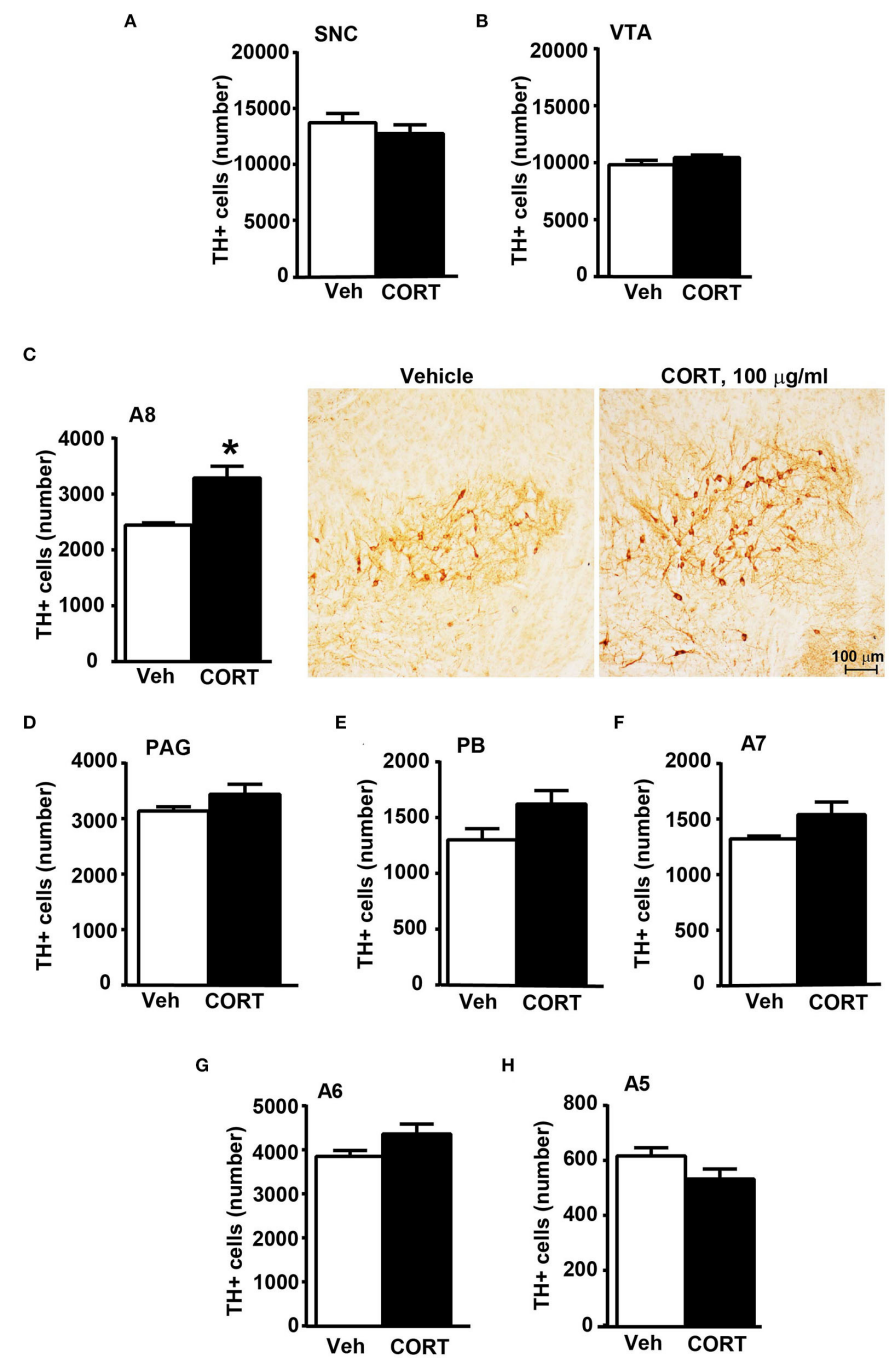
Chronic treatment with corticosterone increases the number of TH-positive cells in A8 inside the cranial mouse brainstem reticular formation. The number of TH positive cells in SNC and VTA in the midbrain of mice subjected to chronic treatment for 5 weeks with vehicle (ethanol 0.66%) or corticosterone (CORT, 100 μg/ml) is shown in **(A,B)**, respectively [**(A)** vehicle 13721 +/– 890, CE: 0.093 +/– 0.005, CV: 0.184; CORT 12764 +/– 833, CE: 0.093 +/– 0.003, CV: 0.173; **(B)** vehicle 9822 +/– 406, CE: 0.085 +/– 0.003, CV: 0.117; CORT 10459 +/– 225, CE: 0.080 +/– 0.003, CV: 0.057]. **(C)** Stereological counting of TH immunoreactive cells in A8 of mice subjected to the same experimental conditions (vehicle 2445 +/– 40, CE: 0.108 +/– 0.002, CV: 0.047; CORT 3287 +/– 225, CE: 0.100 +/– 0.002, CV: 0.182). Representative images showing the robust increase of TH immunoreactive cells in A8 of corticosterone-treated mice are shown in the right part of **(C)**. The number of TH-positive cells in PAG, PB, A7, A6, and A5 in the cranial brainstem of mice subjected to the same experimental conditions is shown in **(D–H)**, respectively. Values are means +/– S.E.M. of nine (vehicle) and eight (CORT) mice per group (one mouse in the group of corticosterone-treated mice was eliminated as a non-responder). ^*^p < 0.05 Unpaired two-tailed Student *t*-test. CE, Coefficient of Error; CV, Coefficient of Variation. [**(D)** vehicle 3139 +/– 81, CE: 0.101 +/– 0.002; CORT 3440 +/– 194, CE: 0.093 +/– 0.03, CV: 149; **(E)** vehicle 1302 +/– 106, CE: 0.134 +/– 0.006, CV: 0.230; CORT 1623 +/– 128, CE: 0.119 +/– 0.005, CV: 0.209; **(F)** vehicle 1323 +/– 25, CE: 0.163 +/– 0.003, CV: 0.054; CORT 1541 +/– 122, CE: 0.146 +/– 0.007, CV: 0.210; **(G)** vehicle 3853 +/– 139, CE: 0.099 +/– 0.002, CV: 0.102; CORT 4360 +/– 240, CE: 0.085 +/– 0.003, CV: 0.102; **(H)** vehicle 616 +/– 32, CE: 0.237 +/– 0.007, CV: 0.146; CORT 533 +/– 39, CE: 0.244 +/– 0.009].

Stereological quantification performed in catecholamine nuclei from the anterior brainstem indicates a significant increase in the number of TH-positive cells in response to treatment with corticosterone within the RRF (A8, as shown in the graph and representative pictures of Figure 9C). In the SNC, VTA, PAG, PB, A7, and A6, an increase was steady although non-significant (Figures 9A,B,D–G, respectively). On the contrary, the A5 shows a decrease in TH-positive cells (Figure 9H).

### In the caudal brainstem, TH-immunopositive cells increase significantly within area postrema

**Figure 10:**
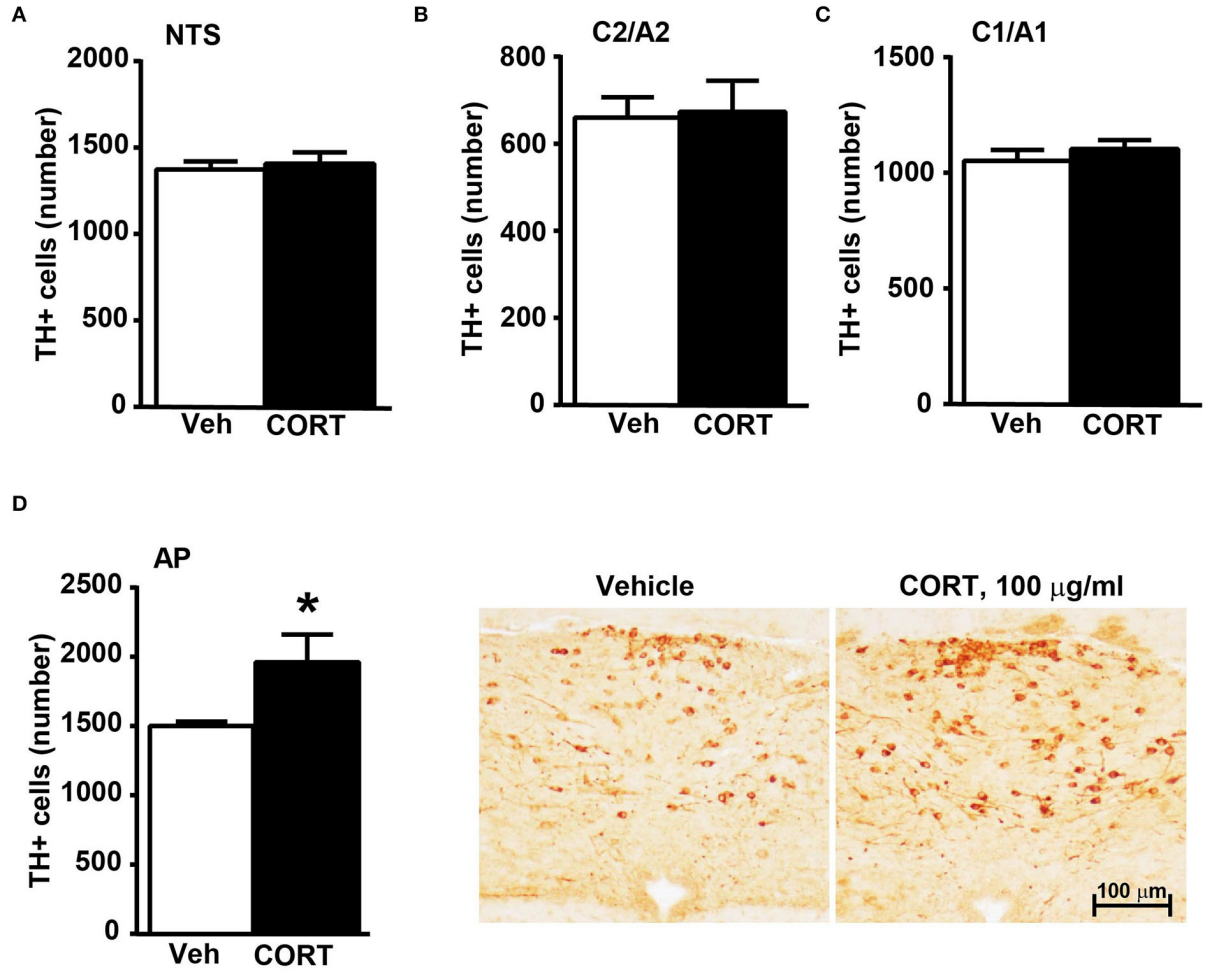
Chronic treatment with corticosterone increases the number of TH-positive cells in AP in the caudal mouse brainstem reticular formation. The number of TH-positive cells in NTS, C2/A2, C1/A1, and AP of mice chronically treated for 5 weeks with vehicle (ethanol 0.66%) or corticosterone (CORT, 100 μg/ml) is shown in **(A–D)**, respectively. Values are means +/– S.E.M. of nine (vehicle) and eight (CORT) mice per group (one mouse in the group of corticosterone-treated mice was eliminated as a non-responder). ^*^p < 0.05 Unpaired two-tailed Student *t-*test. CE, Coefficient of Error; CV, Coefficient of Variation. [**(A)** vehicle 1374 +/– 50, CE, 0.129 +/– 0.02, CV: 0.103; CORT 1407 +/– 70, CE: 0.126 +/– 0.002, CV: 131; **(B)** vehicle 661 +/– 49, CE: 0.099 +/– 0.002, CV: 0.209; CORT 674 +/– 76, CE: 0.097 +/– 0.002, CV: 0.298; **(C)** vehicle 1052 +/– 50, CE: 0.090 +/– 0.001, CV: 0.136; CORT 1104 +/– 41, CE: 0.090 +/– 0.002, CV: 0.097; **(D)** vehicle 1502 +/– 31, CE: 0.124 +/– 0.002, CV: 0.059; CORT 1961 +/– 215, CE: 0.112 +/– 0.005, CV: 0.291]. Representative images showing the robust increase of TH immunoreactive cells in AP of corticosterone-treated mice are shown in the right part of **(D)**.

Stereological counting of TH-positive cells in response to treatment with corticosterone in C1/A1 (Figure 10C) shows an increase that remains non-significant. Similarly, non-significant variations are detected within either C2/A2 (Figure 10B) or NTS (Figure 10A). In contrast, we found a significant increase in the number of TH immunoreactive cells in AP of mice chronically administered corticosterone compared with control vehicle-administered mice (Figure 10D).

### Correlation analysis between the number of TH-positive cells and glucose tolerance or body weight changes in response to treatment with vehicle or corticosterone

**Figure 11:**
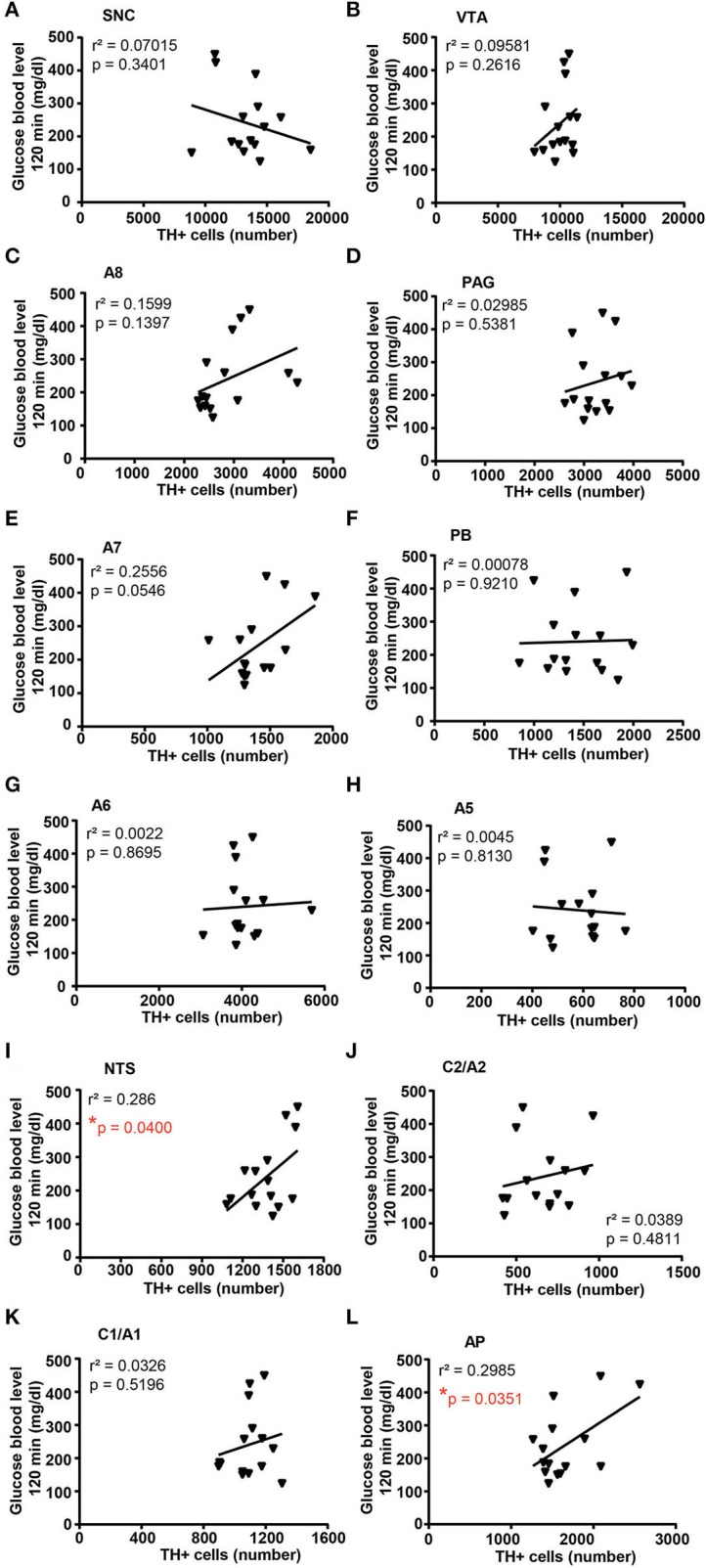
There is a positive correlation between the number of TH-positive cells in NTS and AP and glucose intolerance. Correlation analysis between the number of TH positive cells in SNC, VTA, A8, PAG, A7, PB, A6, A5, NTS, C2/A2, C1/A1, and AP and the glucose blood level monitored 120 min after a bolus of glucose (glucose 20% in 0.9% NaCl, i.p.) in mice treated for 4 weeks with vehicle (0.66% ethanol, *N* = 8; in the group of vehicle-treated mice, one mouse was excluded as not-responder to the glucose bolus) or corticosterone (100 μg/ml in the drinking water, *N* = 7; in the group of corticosterone-treated mice, one mouse was excluded as non-responder to the glucose bolus and one mouse was excluded as not-responder to corticosterone treatment) is shown in **(A–L)**, respectively. *p < 0.05, Pearson correlation test.

**Figure 12:**
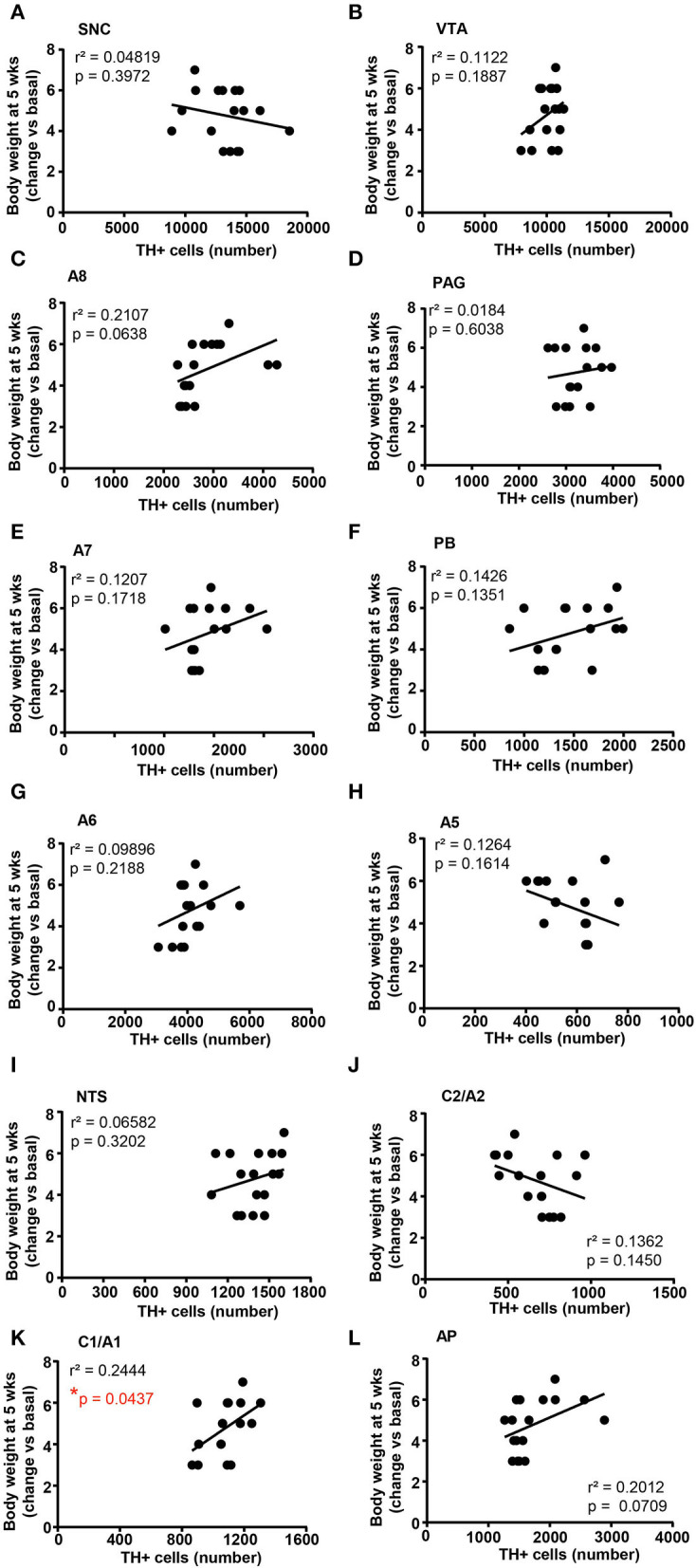
There is a positive correlation between the number of TH-positive cells in C1/A1 and the body weight increase. Correlation analysis between the number of TH positive cells in SNC, VTA, A8, PAG, A7, PB, A6, A5, NTS, C2/A2, C1/A1, and AP and the body weight changes vs. basal (before treatment) in mice treated for 5 weeks with vehicle (*N* = 9) or corticosterone (*N* = 8; 1 mouse was excluded as not-responder to the corticosterone treatment) is shown in **(A–L)**, respectively. *p < 0.05, Pearson correlation test.

A correlation analysis was carried out between the number of TH-positive cells in each catecholamine nucleus of the brainstem reticular formation and parameters of glucose tolerance [the glucose blood levels detected 120 min after a bolus of glucose administered after 4 weeks of treatment with vehicle or corticosterone (Figure 11)] or body weight changes [body weight changes following 5 weeks of treatment with vehicle or corticosterone compared with respective values measured before the treatment (Figure 12)].

Concerning glucose tolerance, a positive correlation was selectively found between the number of TH-positive cells and glucose blood levels in NTS and AP (Figures 11I,L, respectively), while no significant correlation was detected in SNC, VTA, A8, PAG, A7, PB, A6, A5, C2/A2, and C1A1 (Figures 11A–J,K; respectively).

When the correlation analysis was carried out for the body weight changes, we selectively found a positive correlation between the number of TH-positive cells in the A1/C1 group and the increase in body weight (Figure 12K). No correlation was found between the number of TH-positive cells and the body weight changes in SNC, VTA, A8, PAG, A7, PB, A6, A5, NTS, C2/A2, and AP (Figures 12A–H,J,L, respectively).

## Discussion

The present *ex vivo* study indicates that chronic administration of corticosterone significantly increases the number of catecholamine cells within two specific nuclei, the RRF and AP, posed respectively, at the rostral and caudal poles of the brainstem reticular formation.

These findings are in line with data showing that glucocorticoid receptor stimulation increases TH gene transcription (Hagerty et al., 2001). The promoter region of the TH gene contains glucocorticoids responsive element (GRE) (Hagerty et al., 2001) and elevated glucocorticoid levels accelerate both release and turnover of brain catecholamines (Abercrombie et al., 1989; Finlay et al., 1995; Sullivan, 2004; Kvetnansky et al., 2009). Chronic stress exposure activates catecholamine neurons (Mamalaki et al., 1992; Watanabe et al., 1995; Rusnak et al., 2001; Flugge et al., 2004) and long-term, reiterated stress exposure sensitizes the increase of TH mRNA levels in response to novel stressors (Serova et al., 1998, 1999; Rusnak et al., 2001; Tumer et al., 2001; Kvetnansky et al., 2003). However, during prolonged stress, the hyperactive state of catecholamine neurons may deplete catecholamine stores, which eventually may reduce the activity of catecholamine neurons (Loughlin et al., 1986; Valles et al., 2006). Moreover, alterations of catecholamine systems during chronic stress may contribute to neurodegeneration. Stress-related catecholamine alterations might accelerate neuronal degeneration by increasing the formation of toxic dopamine and norepinephrine by-products (Martinez-Vicente et al., 2008; da Luz et al., 2015; Sugama et al., 2016; Goldstein and Kopin, 2018; Kang et al., 2020; Fornai and Puglisi-Allegra, 2021).

The present findings provide a quantitative measurement of 12 TH-expressing nuclei, being analyzed by automated stereology, in brainstem reticular formation of mice subjected to chronic administration of corticosterone. The increase in TH-positive cells was significantly and selectively identified within two catecholamine cell groups, AP and RRF. While the increase in A1/C1, although non-significant *per se*, significantly correlates with the increased body weight of C57Bl/6 mice.

Remarkably, the increase of TH-positive cells within AP is significantly correlated with the onset of reduced tolerance to glucose in line with specific control of AP on those neurons in the dorsal vagal complex which innervates the pancreas (Loewy et al., 1994). The present study also indicates an increase of TH-positive cells within A1/C1, which, although is non-significant, is significantly correlated with an increase in the body weight of corticosterone-treated mice. The present findings detail and extend previous findings we obtained *in vitro*, which documented corticosterone-induced increased expression of catecholamine markers within organotypic cell cultures dissected from the caudal brainstem (Busceti et al., 2019).

Thus, the present data obtained in the whole brainstem indicates that chronic corticosterone elevates TH-positive cells within the RRF within the rostral brainstem, which differs from organotypic cell cultures where no effect was detected neither in slices from the pons nor the mesencephalon (Busceti et al., 2019).

Despite a significant increase in the number of TH-positive cells in A8, the greatest dopaminergic nuclei (A9 and A10) do not show any increase. These data suggest that there is no correlation between the alterations induced in response to chronic exposure to corticosterone and a specific neurotransmitter in all nuclei. The effect is rather confined to specific nuclei.

Remarkably, in the present *ex vivo* study, the increase in TH-positive cell bodies is selective for two nuclei, although it occurs non-significantly for most TH-positive nuclei, where the number of TH-positive cells surpasses those counted from the control brain. This trend is magnified compared to that documented in organotypic slices of the anterior brainstem where the overall amount was not significantly different (Busceti et al., 2019).

This widespread increase in TH immunoreactivity in the cranial brainstem does not occur in A5, where the trend is opposite to the other nuclei.

Although both AP and the RRF develop a significant increase of TH-positive neurons, only the increase in AP but not the increase in RRF correlates with corticosterone-induced glucose intolerance. It is expected that further studies will allow us to disclose a causal relationship between the increase of TH-positive cells within RRF (A8) and the development of other alterations associated with Cushing's syndrome, such as anxiety, aggressiveness, or sleep-waking cycle dysfunction. RRF is involved in a number of behavioral states and a number of axons connect A8 dopaminergic neurons with neurons in the amygdala (Wallace et al., 1989).

The amygdala, which is in close connection with the medial prefrontal cortex (mPFC) controls emotional responses, such as fear and anxiety (Sotres-Bayon and Quirk, 2010; Kumar et al., 2014; Likhtik et al., 2014; Bukalo et al., 2015), which characterize Cushing's syndrome. Corticosterone-induced emotional changes may likely be partly related to its effects on RRF (A8) neurons.

In fact, mPFC exerts an inhibitory control on the amygdala activity, thus controlling emotional behaviors (Rosenkranz and Grace, 2001; Quirk et al., 2003; Rosenkranz et al., 2003; Motzkin et al., 2015). Evidence exists that chronic corticosterone treatment in mice produces defective prefrontal inhibitory control of the amygdala (Liu et al., 2020) fostering anxiety and depression (Quirk and Gehlert, 2003; Correll et al., 2005; Rauch et al., 2006). Thus, corticosterone may produce a dual effect *via* RRF and mPFC, which synergize to produce anxiety and mood disorders. Specific behavioral investigations are needed to address this point.

Concerning the caudal part of the brainstem reticular formation, AP features a significant increase in the number of TH-positive in response to chronic treatment with corticosterone. This is fascinating since AP provides a selective control to the vagal efferent, which innervates the pancreas (Loewy et al., 1994). This is in line with altered tolerance to glucose occurring in these mice, which, in turn, significantly correlates with the increase in TH-positive cells within AP.

This is in line with the role of TH-positive cells within AP and its rostral branching within NTS as gluco-sensing neurons (Roberts et al., 2017). In detail, high glucose levels increase the firing of catecholamine neurons within AP and NTS by increasing spontaneous glutamate inputs (Roberts et al., 2017). This provides a mechanism by which changes in glucose could impact catecholamine neurons in the medullary reticular formation, thus controlling cardiovascular, respiratory, and gastrointestinal systems (Simon et al., 1985; Kubo et al., 1990; Itoh and Buñag, 1993; Schild et al., 1994; Saper et al., 2002; Olson et al., 2006). The present correlation data suggest that high plasmatic levels of glucocorticoids through increasing the number of catecholamine neurons in the AP may provide a neuroanatomical substrate extending beyond glucose intolerance.

In keeping with the caudal brainstem, considering the C1/A1 region, the increase in TH-positive cells is not significant; however, a significant correlation was measured between the number of TH-positive cells and increased body weight. This is very intriguing since these neurons of the ventrolateral medulla project to the paraventricular hypothalamic areas and are involved in feeding behavior (Rinaman, 1999; Gaykema et al., 2007). This suggests a neural basis to explain why glucocorticoids alter feeding activity, which, in turn, may contribute to metabolic alterations, fat re-distribution, and glucose intolerance concomitant to an increase in body weight, which occurs in Cushing's syndrome.

## Conclusion

Chronic treatment with corticosterone induces *in vivo* a significant and selective alteration of TH-positive neurons within two nuclei placed in the lateral column of the brainstem reticular formation. These alterations significantly correlate with the selective domain of Cushing's syndrome. To our knowledge, this is the first study that provides a potential anatomical basis that may underlie specific symptoms occurring in Cushing's syndrome.

## Data availability statement

The raw data supporting the conclusions of this article will be made available by the authors, without undue reservation.

## Ethics statement

The animal study was reviewed and approved by Neuromed Institute Ethical Committee Ministry of Health (Authorization #1132/2016-PR).

## Author contributions

CLB performed immunohistochemical analysis, statistical analysis, and wrote the manuscript. DB performed immunohistochemical analysis and stereological counting. PD performed glucose tolerance test. MS and MF revised the manuscript. SP-A, FN, and FF supervised research and revised the manuscript. All authors contributed to the article and approved the submitted version.

## Funding

This work was supported by a grant from the Italian Ministry of Health (Ricerca Corrente to FF through IRCCS Neuromed).

## Conflict of interest

The authors declare that the research was conducted in the absence of any commercial or financial relationships that could be construed as a potential conflict of interest.

## Publisher's note

All claims expressed in this article are solely those of the authors and do not necessarily represent those of their affiliated organizations, or those of the publisher, the editors and the reviewers. Any product that may be evaluated in this article, or claim that may be made by its manufacturer, is not guaranteed or endorsed by the publisher.
